# Retraction: Wavelength modulation of ZnO nanowire based organic light-emitting diodes with ultraviolet electroluminescence

**DOI:** 10.1039/d0ra90138e

**Published:** 2021-01-04

**Authors:** Runze Chen, Chuan Liu, Kyeiwaa Asare-Yeboah, Ziyang Zhang, Zhengran He, Yun Liu

**Affiliations:** Leicester International Institute, Dalian University of Technology Panjin City 124221 China; State Key Laboratory of Structural Analysis for Industrial Equipment, Dalian University of Technology Dalian 116024 China; Department of Electrical and Computer Engineering Penn State Behrend Erie PA 16563 USA; Department of Electrical Engineering, Columbia University New York City NY 10027 USA; Department of Electrical and Computer Engineering, The University of Alabama Tuscaloosa AL 35487 USA zhe3@crimson.ua.edu; Department of Physics, Dalian University of Technology Dalian 116024 China liuyun89@dlut.edu.cn

## Abstract

Retraction of ‘Wavelength modulation of ZnO nanowire based organic light-emitting diodes with ultraviolet electroluminescence’ by Runze Chen *et al.*, *RSC Adv.*, 2020, **10**, 23775–23781, DOI: 10.1039/D0RA04058D.

We, the named authors, hereby wholly retract this *RSC Advances* article due to a calibration error in the CVD tool used when fabricating the inverted UV-OLED devices. As a result, the subsequent data collected is not reliable and the conclusions of this paper are not supported.

Signed: Runze Chen, Chuan Liu, Kyeiwaa Asare-Yeboah, Ziyang Zhang, Zhengran He and Yun Liu

Date: 29^th^ November 2020

Retraction endorsed by Laura Fisher, Executive Editor, *RSC Advances*

## Supplementary Material

